# Have you “involution” today—Competition psychology scale for college students

**DOI:** 10.3389/fpsyg.2022.951931

**Published:** 2022-10-20

**Authors:** Yisi Liu, Yanli Tu, Hao Yang, Jie Gao, Yun Xu, Qiwei Yang

**Affiliations:** ^1^School of Psychology, Chengdu Medical College, Chengdu, China; ^2^Sichuan Applied Psychology Research Center, Chengdu Medical College, Chengdu, China

**Keywords:** hypercompetitive attitude, competitive motivation, personal development competitive attitude, competitive interpersonal relationship, competition psychology, involution

## Abstract

In order to investigate the competitive psychology of college students in the current context of fierce social competition, this study compiled a competition psychology scale for college students [i.e., the Competition Psychology Scale for College Students (CPS-CS)]. The scale was administered online to 628 university students in different regions of China. After item analysis, reliability analysis, and validity analysis, a 6-item scale was finally formed. CPS-CS contains four dimensions: hypercompetitive attitude, competitive motivation, personal development, competitive attitude, and competitive interpersonal relationships. The reliability and validity of the CPS-CS developed in this research meet the requirements of psychometrics.

## Introduction

Currently, society is very competitive. In recent years, a new buzzword has emerged in China: “involution” (translated into Chinese as “neijuan”). The term is originally an anthropological term for a phenomenon in which a pattern reaches a certain form and has no way of either stabilizing or transforming into a new form but only continues to become more complex internally (Geertz, 1963). Many Chinese higher education students use the term “involution” to refer to irrational internal competition or “be volunteered to” competition. In China now, involution refers to the phenomenon of peers competing to work harder for limited resources, resulting in a decline in the individual’s “effort-to-reward” ratio. It can be seen as an “inflation” of effort. Later, the term “anti-involution” emerged, meaning opposition to excessive and irrational competition. The rise of these buzzwords has fully reflected the fierce and complex social competition, people’s anxiety and helplessness about competition, and their emotions and attitudes toward not giving up and continuing to struggle. As a major group in society, college students face competition from academics, clubs, and interpersonal relationships in school on the one hand and the pressure of competing with others in society and the workplace on the other. On college campuses, students need to compete to gain knowledge, skills, education, achievements, and many other experiences and resources necessary for growth. At the same time, the employment problem faced by college students after graduation is also a hot spot that society cannot ignore for a long time. Therefore, this study takes college students as the research group to study the competitive psychology of college students.

Competition is a process in which different subjects compete for limited resources. According to previous research and the competitive behaviors of college students in real life, the factors influencing the competitive psychology of college students might mainly include two aspects. One is the individual competitiveness of college students’ personality traits, including their attitudes, motivations, emotions, and affections in competition. The other one is the interpersonal relationships of college students in competition.

Many scholars have explored the structure of competitive psychology and the factors influencing it. Martin and Larsen developed the Competitive Cooperation Attitude Scale (CCAS) by collecting data on the psychology of competition in a game-related behavioral experiment (Martin and Larsen, 1976). The concept of hypercompetitive attitudes was introduced by Ryckman and his colleagues based on Horney’s theory of pathological competitive behavior, and they developed the Hypercompetitive Attitude Scale (HCA), which adds another dimension to the measurement of competitive psychology (Ryckman et al., 1990). Later, they proposed another concept: personal development and competitive attitude. They created the Personal Development Competitive Attitude Scale (PDCA) to measure this trait (Ryckman et al., 1996). The following research shows that hypercompetitive and personal developmental and competitive attitudes, as measured by the two previously mentioned scales, are two different traits, meaning that they are mutually independent dimensions of individual competitive psychology (Ryckman et al., 1997). Guopeng Chen revised the HAS, and the PDCA developed a Chinese version of the Competitive Attitude Scale. He found that competitiveness was often influenced by the strength of an individual’s own motivation (Chen et al., 2003).

The researcher identified the factors of competitive motivation. The five factors were satisfaction, which comes from improving one’s performance, desire to win, motivation to put forth effort in competitive situations, satisfaction that comes from performing well, and preference for difficult tasks (Franken and Brown, 1995). In a study of athletes’ competition-related motivations, Willis found that power, achievement, and fear of failure combined to influence athletes’ competitiveness (Willis, 1982). In China, some scholars have also included competitive motivation in their studies of competitive psychology. For example, the Competition Psychology Inventory for College Students was developed by Cen (2009), and the Psychology of Competition Questionnaire for Secondary School Students was developed by Zhou et al. (2007).

In addition, the emotions and feelings accompanying competition cannot be ignored. Research shows that cultivating a good competitive mentality can not only improve or even eliminate negative moods and emotional experiences such as depression and anxiety but also stimulate the potential of individuals to be more proactive in adapting to the current stressful and intense social environment (Chen, 2000). Studying emotions before, during, and after the competition has essential theoretical and practical implications. They are thought to objectively or subjectively influence competition performance (Lane and Terry, 2000; Hanin, 2007). In the past, research on the relationship between emotions and competition has mostly been limited to anxiety (Jones, 1995; Burton and Naylor, 1997). Some scholars point out that attention should be paid to the broader underlying emotions of competition, such as depression, fear, apprehension, and so on (Hanin, 2007). However, due to the strong practical application properties of competing anxiety emotions, there is still a tendency in the academic community to ignore others and focus too much on anxiety.

From the above studies, we can find that most scholars in previous studies on competitive psychology have explored competitiveness as a personality trait, but the relationship between competitiveness and individual personality structure has not been conclusively established. The current studies are fragmented. Furthermore, competition is an inter-individual behavior, and interpersonal relationships in the competition are highly correlated with the individual’s competitive psychology. Griffin-Pierson developed the Interpersonal Competitiveness Questionnaire (CQ) (Griffin-Pierson, 1990). Most of the previous studies ignored that competition is an interpersonal behavior and separated competitiveness as a trait of individuals from interpersonal relationships, taking into account both individual and interpersonal perspectives. At the same time, as the main group of people competing in contemporary society, the research on their competitive psychology is still incomplete.

In summary, this study explores the structure of competitive psychology among college students, uses methods such as factor analysis to develop a scale adapted to the competitive psychology of Chinese college students, and analyzes the reliability and validity of the scale to enrich the research in the field of competitive psychology.

## Scale construction and development

### Structure constructing

The competitive psychology of college students includes individual competitiveness and interpersonal relationships and needs to be reflected in the process of specific behavioral activities. The development of CPS-CS follows the principle of using theory as a guide, synthesizes scientific research papers that focus on competition psychology, and constructs the dimensions in terms of attitudes, interpersonal relationships, motivation, and emotions of competition.

To make the structural dimensions of competitive psychology more consistent with the characteristics of contemporary Chinese college students, we randomly interviewed 69 college students across China based on some theoretical support. The questions were semi-structured to expand and enrich the content of the scale: “What words or phrases come to mind when you think of the competition?”

With reference to previous results and open-ended research surveys by previous authors, this research constructed and developed the initial CPS-CS. The scale contains a complete and clear structure of the competitive psychological system. The initial scale includes five dimensions as follows: (1) hypercompetitive attitude (HCA): compete, win or avoid defeat at any cost, even to the point of excluding others or harming their interests, and as a means of maintaining or enhancing a sense of self-worth; (2) personal development competitive attitude (PDCA): people with this attitude value the pleasure of the task and the process rather than the winning result, and they compete with an orientation to developing their abilities and potential; (3) competing emotions and affections (CEA): various expressions of positive or negative emotions and affections due to competition; (4) competitive motivation (CM): different levels of motivation have an impact on individuals’ competitive psychology, behavior, and strategies; and (5) competitive interpersonal relationships (CIR): to a certain extent, the state of interpersonal tension can explain the high or low competitiveness of individuals themselves.

### Item generation

The initial CPS-CS includes five dimensions with a total of 42 questions. The items were randomly arranged, and some were scored in the reverse direction to prevent stereotypes in the process of answering the questions and reducing measurement error. CPS-CS asks the participant to choose the option that matches his or her true situation—that is, the degree to which the item’s description matches the participant’s. CPS-CS is scored on a five-point Likert scale (1 = Strongly disagree, 2 = Disagree, 3 = Sometimes disagree, sometimes agree, 4 = Agree, 5 = Strongly agree).

### Participants

The scale administration was conducted through an online web-based questionnaire (wenjuan.com). A convenience sampling method was used to measure 641 college students in China, and 628 valid questionnaires were finally obtained after excluding 13 unqualified results. Among them, 251 were male students, and 377 were female students. The collected data were statistically analyzed using SPSS 21.0 and Amos 24.0.

## Results

### Structure analysis

#### Item analysis

The collected data were subjected to item analysis (IA), including the calculation of the item’s discriminatory and critical values. Item discrimination was first examined. The top and bottom 27% of the total scale score were used as the high and low subgroups. The differences between the two groups on each item were observed by independent samples *t*-test and items with a significant *p*-value of <0.05 needed to be deleted. This process removes eight items. Next, item-total statistics were used to check the critical value of the items, and items with a critical value below 0.2 should be deleted. A total of 24 items remained in the end.

#### Exploratory factor analysis

The sample data of the participants were randomly divided into two groups, A and B. Exploratory factor analysis (EFA) was performed on the group A data (*n* = 310). The Kaiser–Meyer–Olkin (KMO) measure was 0.823, indicating suitability for factor analysis (FA). According to the criterion that the characteristic root must be greater than 1, there are seven extractable factors. Among them, the variance contribution of the first four factors was 44.35%, and the average variance contribution of each factor was over 8%. From the fifth factor onward, the average variance contribution of the first five factors was less than 7%. The screen test shows that the inflection point exists on the fourth factor. In summary, CPS-CS is suitable for extracting four factors.

Factor analysis was performed again using principal component analysis (PCA) and the varimax-rotation method (VRM). Items with a commonality below 0.30, factor loading below 0.40, or a difference cross-loaded onto other factors larger than 0.20 would be removed. According to the criteria for reduction, eight items were removed, and 16 remained. EFA again for sample A (*n* = 310). The KMO measure was 0.809, and Bartlett’s test result was 1,167.61 (*p* < 0.00). As Table 1 displays, there are four factors with eigenvalues greater than 1, and each item has a clear factor attribution. Based on the content of the items contained within the factors, the four factors were defined as follows: (1) hypercompetitive attitude (HCA): reflects that to win the competition, people may behave and think in a way that excludes others or harms others’ interests; they want to ensure that they can win; (2) competitive motivation (CM): reflects the effects of different levels of motivation on human competitive psychology, behavior, and strategy; (3) personal development competitive attitude (PDCA): reflects the behavior and ideas of individuals who compete to develop their abilities and potential; (4) competitive interpersonal relationship (CIR): reflects the influence of interpersonal relationships in the individual’s competition on individual competitiveness. The above four factors cumulatively explained 53.7% of the variance.

**TABLE 1:** Exploratory factor analysis of CPS-CS.

Item	HCA	CM	PDCA	CIR
5. I am willing to sacrifice the interests of others to succeed.	0.579			
13. When I succeed, I think people get jealous of me.	0.601			
21. I secretly rejoice in the failure of my competitors.	0.68			
35. Competition creates enemies.	0.649			
38. If I can somehow interfere with my opponent to gain a competitive advantage, I will do so.	0.711			
7. Without the challenge of competition, I may not have discovered that I have certain potential and talents.		0.744		
16. I like competition because it gives me the opportunity to discover my own abilities.		0.54		
18. I value competition because it allows me to become the best I can be.		0.651		
22. I will study hard to get an edge over the competition.			0.79	
29. Competition can improve my social skills.			0.494	
40. I will put a lot of effort into being successful in the competition.			0.805	
34. I find competition attractive because it can test my ability.			0.506	
39. I like competition because it often motivates me to bring out the best in myself, not because it gives me a sense of superiority.			0.604	
6. Even in an environment where I don’t have to compete, I want to compete with others.				0.568
31. I participate in the competition because I like the feeling of competing with others.				0.808
43. Regardless of the outcome, competition excites me.				0.698
Eigen values	2.41	1.70	2.55	1.95
% Variance	15.05%	10.61%	15.93%	12.16%

HCA, hypercompetitive attitude; CM, competitive motivation; PDCA, personal development competitive attitude; CIR, competitive interpersonal relationship. **p < 0.01.

#### Confirmatory factor analysis

**FIGURE 1:**
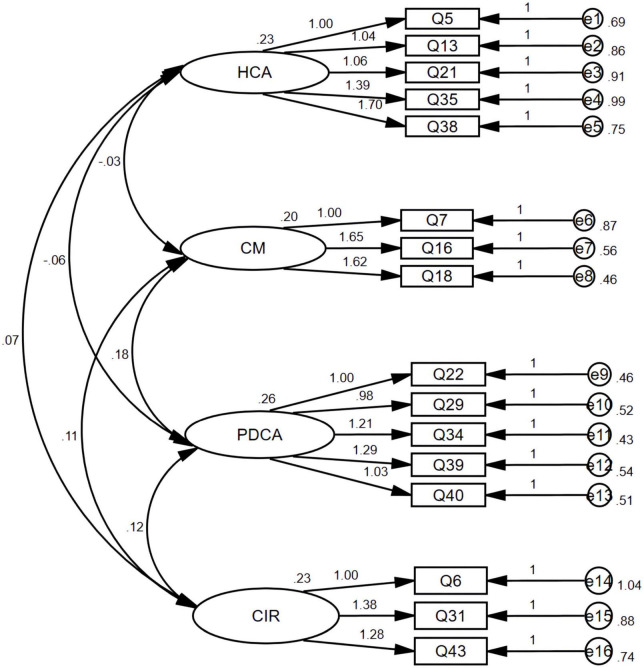
Confirmatory factor analysis of CPS-CS, the four-factor model.

Confirmatory factor analysis (CFA) was performed on sample B data (*n* = 336). The four-factor model fits the data better (see Figure 1). As shown in Table 2, each model index meets the statistical requirements.

**TABLE 2:** Confirmatory factor analysis of CPS-CS, indexes of the model.

Fit statistics	χ^2^	df	χ^2^/df	RMSA	CFI	GFI	IFI	AGFI	RMR
No.	188.962	98	1.928	0.053	0.914	0.934	0.916	0.909	0.067

**p < 0.01.

### Correlation between dimensions and total score

Table 3 indicates HCA had a non-significant correlation with CM (*r* = -0.059, *p* < 0.01). The Pearson correlation coefficients between the remaining dimensions presented expected associations with a significant coefficient of correlation (*r* ranged from 0.158 to 0.60, *p* < 0.01). All four dimensions are significantly correlated with the total score (*r* ranged from 0.442 to 0.715, *p* < 0.01).

**TABLE 3:** Correlations among dimensions of CPS-CS.

	HCA	CM	PDCA	CIR
HCA	1			
CM	−0.059	1		
PDCA	0.185**	0.600**	1	
CIR	0.158**	0.395**	0.375**	1
CPS-CS	0.442**	0.704**	0.686**	0.715**

HCA, hypercompetitive attitude; CM, competitive motivation; PDCA, personal development competitive attitude; CIR, competitive interpersonal relationship; CPS-CS, Competition Psychology Scale for College Students. **p < 0.01.

### Reliability and validity tests

#### Reliability analysis

In terms of the four dimensions, Cronbach’s α was 0.654 for HCA, 0.655 for CM, 0.776 for PDCA, and 0.532 for CIR. Cronbach’s α for CPS-CS was good, α = 0.718. ω reliability was 0.661 for HCA, 0.680 for CM, 0.780 for PDCA, 0.539 for CIR, and 0.713 for CPS-CS. Additionally, the test-retest reliability was significant, at *r* = 0.601.

### Validity analysis

Competition Psychology Scale for College Students was constructed with reference to existing studies and based on a certain theoretical framework. The items were made better by the interview method. The initial scale was guided and judged by professional psychology professors. In summary, CPS-CS has good content validity.

To further examine the criterion validity, correlational analysis was conducted to explore the association between the CPS-CS and the Consequences of perfectionism (COPS), Connor-David Resilience Scale (CD-RISC), and Multidimensional Competitive Orientation Inventory (MCOI). The results showed a moderate correlation coefficient with the total score of the COPS (*r* = 0.514, *p* < 0.01). The four dimensions of CPS-CS presented a low correlation with the total score of the COPS (*r* ranged from 0.242 to 0.380, *p* < 0.01). The results of the correlation analysis with the CD-RISC found a significant moderate or low correlation coefficient with CPS-CS (*r* = 0.392, *p* < 0.01) and the four factors (*r* ranged from -0.111 to 0.494, *p* < 0.01). The analysis also indicated a low correlation between the total score of MCOI and CPS-CS, *r* = 0.337 (*p* < 0.01). Besides, CM and PDCA showed no statistically significant correlations with the MOCI. These data demonstrate that CPS-CS measures relatively independent, competitive psychological traits. The details of criterion validity are presented in the Supplementary Tables.

## Discussion

The intensity of social competition is gradually increasing with the rapid development of the social economy and the continuous improvement of the material level. This intense competition is especially prominent and strong in colleges. Based on the literature study, this research used questionnaires and expert analysis to construct the structural framework, dimensions, and items of CPS-CS and conducted FA reliability tests, and validity tests.

Based on the literature study, this research used questionnaires and expert analysis to construct the structural framework, dimensions, and items of CPS-CS. Compared with the existing results, the study group of CPS-CS is more targeted. At a time when “involution” is a buzzword, universities are certainly on the cusp of the “involution” trend. The CPS-CS is designed for a group of Chinese university students, and the competitive psychology of this group is systematically investigated. The CPS-CS is compiled, and the discrimination, reliability, and validity of the scale are analyzed through a large sample survey.

The results of the analysis showed that the reliability and validity of CPS-CS reached an acceptable level and could predict the competitive psychology traits of college students. The formal CPS-CS has few items and has the advantage of being simple and easy to administer. From the FA of the 16 items, it is clear that the structure of competition psychology among college students assumed in this study is feasible. However, some dimensions were low-level and consisted of only a few items. Therefore, they still need to be more fully exploited.

The initial scale listed 43 items. After IA, the unqualified items were deleted. The EFA of the remaining 23 items demonstrated that the number of suitable extraction factors was four. A total of seven items were removed in the subsequent principal component analysis as well as in multiple EFAs. This left 16 items divided into four dimensions: hypercompetitive attitudes, competitive motivation, personal development, competitive attitudes, and competitive interpersonal relationships. The CFA results indicated that the model’s overall fit was good. The four-factor model had a suitable fit for the psychological traits to be measured. The internal consistency reliability of CPS-CS (Cronbach’s α = 0.718, *p* < 0.01) and the retest reliability after one week of CPS-CS were good (*r* = 0.601, *p* < 0.01). Validity tests were conducted using COPS, MCOI, and CD-RISC as validity criteria. The Pearson correlation coefficients proved moderate correlations with all three validated criteria, indicating good validity of the CPS-CS.

The initial dimensions of the scale were assumed to be five: hypercompetitive attitudes, competitive motivation, personal development, competitive attitudes, competitive interpersonal relationships, competing emotions, and affections. The results of the FA of CPS-CS suggest that the competitive psychology of college students mainly includes four factors: hypercompetitive attitudes, competitive motivation, personal development, competitive attitudes, and competitive interpersonal relationships. For example, combining previous scholars’ research, Yanyuan Cen et al. conducted an exploratory study on the structure of Chinese competitive psychology, proving that Chinese competitive psychology consists of four dimensions, including benign competitive attitude, hypercompetitive attitude, competitive motivation, and competitive emotion (Cen and Liu, 2018); Willis developed the Sport-Specific Motive Scales to measure motivation in sports competition, including three scales: power, achievement, and fear of failure (Willis, 1982); and Simmons developed the Cooperative/Competitive Strategy Scale to predict the motivation to use cooperative or competitive strategies for success (Simmons et al., 1988). This result is consistent with the theoretical structure. With the exception of competing emotions and affections, the corresponding sub-scales for all four can be found in the formal scale.

Competing emotions and affections were not included in the final factor model. The reasons may be that (1) when the scale was constructed, the affection-related items contained too many different affections, including happiness, anxiety, sadness, depression, anger, fear, and so on, and they are difficult to unify into one dimension; (2) these items are too emotionally charged and may be suggestive to the subjects, making it impossible to accurately measure the target trait; and (3) these items may not be clearly distinguished from items in other dimensions, leading to their removal during FA due to high common loading.

Three scales—COPS, MOCI, and CD-RISC—measuring psychological traits related to competitive psychology were adopted as validity criteria in this study. All of them revealed a significant moderate positive correlation with the total score of this scale. It is generally believed that subjects with perfectionist traits are more willing to engage in competitive activities, and the results obtained from the study indicate just that. COPS demonstrated a significant positive correlation with all four dimensions of CPS-CS. Among the three factors of the CD-RISC: tenacity, strength, and optimism, HCA was negatively correlated with the tenacity and strength factors and insignificantly correlated with the optimism factor, while the other three dimensions all indicated significant positive correlations with the three factors of the CD-RISC. The above results fully demonstrate the good convergent validity of this scale.

In addition, the analysis of CPS-CS showed that the total scale and each dimension had a significant correlation (*r* ranged from 0.442 to 0.715, *p* < 0.01). All dimensions were significantly correlated with each other (*r* ranged from 0.158 to 0.60, *p* < 0.01), except for the correlation between HCA and CM, which was insignificant (*r* = -0.059, *p* < 0.01). In particular, there was a strong positive correlation between CM and PDCA (*r* = 0.6, *p* < 0.01). This is related to the fact that CM includes motivation aimed at the development of individual capabilities. At the same time, it is noteworthy that the CM and the HCA did not reveal a significant correlation result, which implies that the relationship between the motivation to compete and the attitude to compete has some independence. This might be because the items in CM related to a relatively moderate view of personal competence development rather than the more extreme competitive values associated with hypercompetitive. Overall, on the formal scale, using a five-point scale, the higher the score, the more often competitive behaviors are exhibited. Conversely, less competitive behaviors and motivations were exhibited.

There are still some shortcomings in this study. First, the structure of this study may be less stable, which may be due to the lack of clarity in the boundaries of the division between dimensions when the initial scale was developed, resulting in high common loadings for some topics. However, they did not meet the deletion criteria. Second, the initial scale set up several reverse scoring items. However, these items were removed in the subsequent IA and the FA for various reasons. It may be because these items were negative sentences, making it more difficult to understand the questions, and the subjects did not understand the questions to the same extent, thus leading to ambiguous results. Finally, because of the limitation of objective reasons, the number of male and female subjects in the sample was not fully balanced.

In response to the above problems, subsequent studies could make the following improvements: (1) when developing the initial scale, try to clarify the concept, avoid semantic ambiguity, ensure that subjects can clearly understand the meaning of the questions, and try to make each item express the meaning of only one dimension; (2) screening subjects to control various variables as much as possible to ensure the representativeness of the data and the validity of the analysis results; and (3) item response theory can be used to enhance the innovation of scale development. In addition, the social phenomenon of “involution” and the psychology of competition, investigated in this study, are also popular topics of research in economics and mathematics. Several studies have been conducted on related issues (Wang and Hui, 2021; Wang and Szolnoki, 2022). The relevance to these fields can also be explored in our subsequent research.

## Conclusion

This study combined the current “involution” popular topics, based on existing theories and studies and interview methods, to develop CPS-CS and conducted EFA and CFA to verify the reliability and validity of the scale structure. The scale contains four dimensions: hypercompetitive attitude, competitive motivation, personal development, competitive attitude, and competitive interpersonal relationships. The results indicate that CPS-CS meets psychometric requirements and can be used to assess competitive psychology among college students.

## Data availability statement

The raw data supporting the conclusions of this article will be made available by the authors, without undue reservation.

## Ethics statement

The studies involving human participants were reviewed and approved by Institutional Review Board of Chengdu Medical College. Written informed consent for participation was not required for this study in accordance with the national legislation and the institutional requirements. Data collection for this study was conducted on a web-based platform and was anonymous; participants were only required to fill in some demographic information, and no information was registered to identify participants.

## Author contributions

All authors contributed to this article’s data analysis, drafting, revision, agreed on the journal to which the article would be submitted, gave final approval of the version to be published, and agreed to be accountable for all aspects of the work.
