# Supplementary material for: Have you “involution” today—Competition psychology scale for college students
Source: Front Psychol. 2022 Oct 20;13:951931. doi: 10.3389/fpsyg.2022.951931 (PMC9632435; doi:10.3389/fpsyg.2022.951931)
Supplement: Supplementary file 1 [file Table_1.DOCX]

Validity tests were conducted using the Consequences of perfectionism (COPS), Multidimensional Competitive Orientation Inventory (MCOI), and Connor-David Resilience Scale (CD-RISC) as validity criterion. The Pearson correlation coefficients proved moderate correlations with all three validated criterions, indicating good validity of Competition Psychology Scale for College Students (CPS-CS). The results of the analysis are presented in the following tables.

Correlation Analysis with COPS

|  | CPS-CS | HCA | CM | PDCA | CIR |
| --- | --- | --- | --- | --- | --- |
| COPS | 0.514** | 0.242** | 0.321** | 0.380** | 0.349** |
| Consequences of perfectionism (COPS)  **p < .01. | | | | | |

Correlation Analysis with CD-RISC

|  | CPS-CS | HCA | CM | PDCA | CIR |
| --- | --- | --- | --- | --- | --- |
| tenacity |  | -0.079* | 0.309** | 0.434** | 0.348** |
| strength |  | -0.190** | 0.374** | 0.540** | 0.279** |
| optimism |  | 0.002 | 0.193** | 0.294** | 0.140** |
| CD-RISC | 0.392** | -0.111** | 0.345** | 0.494** | 0.325** |
| Connor-David Resilience Scale (CD-RISC)  *p < .05，**p < .01. | | | | | |

Correlation Analysis with MCOI

|  | CPS-CS | HCA | CM | PDCA | CIR |
| --- | --- | --- | --- | --- | --- |
| MCOI | 0.337** | 0.436** | 0.076 | 0.051 | 0.221** |
| Multidimensional Competitive Orientation Inventory (MCOI)  **p < .01. | | | | | |
